# A Secure Long-Range Transceiver for Monitoring and Storing IoT Data in the Cloud: Design and Performance Study

**DOI:** 10.3390/s22218380

**Published:** 2022-11-01

**Authors:** Nurul I. Sarkar, Asish Thomas Kavitha, Md Jahan Ali

**Affiliations:** 1 Department of Computer Science and Software Engineering, Auckland University of Technology, Auckland 1010, New Zealand; 2 School of Engineering, Computer and Mathematical Sciences, Auckland University of Technology, Auckland 1010, New Zealand

**Keywords:** LoRa, transceiver, modules, IoT, LPWAN

## Abstract

Due to the high demand for Internet of Things (IoT) and real-time data monitoring and control applications in recent years, the long-range (LoRa) communication protocols leverage technology to provide inter-cluster communications in an effective manner. A secure LoRa system is required to monitor and store IoT data in the cloud. This paper aims to report on the design, analysis, and performance evaluation of a low-cost LoRa transceiver interface unit (433 MHz band) for the real-time monitoring and storing of IoT sensor data in the cloud. We designed and analyzed a low-cost LoRa transceiver interface unit consisting of a LoRa communication module and Wi-Fi module in the laboratory. The system was built (prototype) using radially available hardware devices from the local electronics shops at about USD 150. The transmitter can securely exchange IoT sensor data to the receiver node at about 10 km using a LoRa Wi-Fi module. The receiver node accumulates the sensor data and stores it in the cloud for processing. The performance of the proposed LoRa transceiver was evaluated by field experiments in which two transmitter nodes were deployed on the rooftop of Auckland University of Technology’s Tower building on city campus (New Zealand), and the receiver node was deployed in Liston Park, which was located 10 km away from the University Tower building. The manual incident field tests examined the accuracy of the sensor data, and the system achieved a data accuracy of about 99%. The reaction time of the transmitter nodes was determined by the data accumulation of sensor nodes within 2–20 s. Results show that the system is robust and can be used to effectively link city and suburban park communities.

## 1. Introduction

LoRa (long-range) communication protocols are the heart of Internet of Things (IoT) applications and connect cloud devices for the real-time monitoring and storage of data for efficiency and productivity purposes. The growing number of IoT sensors makes efficient transmission difficult because of the high-cost infrastructure [1,2,3]. Emerging LoRa technology can be more attractive for long-distance data transmission than the existing wide area network (WAN) communication systems. Most current technologies consume much energy and consequently decrease the battery lifetime of IoT devices. LoRa technology offers simplicity in securing and monitoring IoT sensor data [4]. In addition, this technology can be used for the efficient transmission of IoT sensor data and to enable the system to operate in both outdoor and indoor scenarios [5,6]. Cloud technology delivers a robust infrastructure for controlling information hubs that are tailored to deal with a significant volume of data. Furthermore, a cloud infrastructure can provide storage and surveillance for a large volume of sensor data and can be accessed from anywhere in the world. Existing technologies such as ZigBee [7], NB-IoT [8], and Wi-Fi [6] are not used in the LoRa transceiver system because of data losses, low coverage, and inefficient transmission.

A cost-effective LoRa transceiver system, therefore, is crucial in solving the problems of information transmission in current systems, and such a system assists in the storage and transmission of IoT device information through the execution of a LoRa connector [3,8,9,10]. LoRa systems offer a low-power WAN communication that propose several services for implementation in IoT applications [11]. This LoRa technology can transmit data over a long distance, which is enough for most modern IoT applications [12].

The main objective of this paper is to report on the design (prototype), analysis, and performance testing of a low-cost LoRa transceiver system (433 MHz band) for long-distance communication. Our LoRa transceiver can provide long-range communication without using traditional local and wide area networking technologies. We tested the performance of our proposed LoRa transceiver system in the laboratory as well as using field experiments to quantify the performance gain. Furthermore, we obtained a cloud interface for secure storage, live monitoring, and access to the IoT sensor data from anytime and anywhere across the globe. For the performance testing of the proposed LoRa transceiver, we conducted field experiments at the rooftop and in the laboratory of the Auckland University of Technology Tower building. We also report herein on the data accuracy performance and time efficiency of the proposed LoRa transceiver over 10 km.

Various scientific methods address the issues of data transfer from measurement nodes to destinations using Wi-Fi or ZigBee technologies [13,14,15,16]. A gigahertz band was used in the transceiver system to transfer IoT sensor data over 2.5 km, and the Iridium Satellite Constellation was used in another transceiver for successful communications [8]. We adopted the real-time field measurement method while conducting this research work. The research questions/challenges and research contributions are discussed next.

### 1.1. Research Challenges

In this study, we addressed the following three research questions/challenges.

- Research Question 1: What LoRa infrastructure can be developed to store and retrieve IoT sensor data in the cloud?

To address Research Question 1, we developed a cloud database to transfer IoT sensor data into the database using the Wi-Fi module that we developed and are reporting on in this paper. This Wi-Fi module is integrated into the LoRa system to provide an internet connection to access the cloud services. However, IoT sensor data are stored in the appropriate tables and columns for each sensor node in the database. The system performance is verified by uploading IoT data in the cloud for various climatic and distance conditions.

- Research Question 2: What can be done to monitor real-time IoT sensor data in the system?

To address Research Question 2, we developed a LoRa transceiver system by integrating an LCD unit into the LoRa master receiver node. This allows us to monitor and control real-time IoT data more efficiently. The output data collected from various sensors are verified using an LCD monitor; the data accessibility from the cloud and data synchronization at regular intervals are also verified.

- Research Question 3: What are the main features that affect data loss between the receiver and transmitter end nodes?

To address Research Question 3, herein we identify and discuss the key factors influencing data losses between the receiver and transmitter end nodes, including energy loss, less signal coverage, and low data rates. The proposed LoRa transceiver system provides a secure long-distance communication with low power consumption. For instance, the proposed system consumes about 472 mA of current per year; that is excellent for sustainable communication without any data losses. The signal coverage of the LoRa device is up to 10 km, which is capable of linking city and suburban communities. The data loss in LoRa is less due to its long signal coverage and maximum data rate of 50 kbps.

### 1.2. Research Contribution

The main contributions of this paper are summarized as follows.

- We designed (prototyped) a secure LoRa transceiver system in the laboratory for linking city and park communities at a distance of about 10 km. To this end, we designed, analyzed, and evaluated a LoRa transceiver system.
- We designed and configured a Wi-Fi module to be used in the system for sending and retrieving IoT data to and from the cloud. We evaluated and validated the system performance through various field experiments, including real-time IoT data monitoring and storage in the cloud.
- A secure private cloud database was developed for storing and retrieving of IoT sensor data. The system performance was validated through real-time IoT data captured through various sensors used in the study.

### 1.3. Structure of the Article

The rest of this paper is organized as follows. The related works on LoRa transceivers are presented in Section 2. The LoRa transceiver design and analysis are presented in Section 3. The research methodology is discussed in Section 4. The system evaluation and test results are presented in Section 5; the practical implications are also discussed in this section. Finally, the paper is concluded in Section 6. Table 1 lists the abbreviations used in this paper.

## 2. Related Work

The advances in IoT aim to provide real-time monitoring and intelligent services. The innovative range communication systems such as 4G, 5G, and GPS technologies have been widely used to control and regulate the real-time environments [17,18,19,20]. An outstanding classification of IoT arrangements includes short battery-powered network components such as end nodes, which are equipped with actuators and sensors that wirelessly interact. A common practice implies IoT settings, including the end nodes which collect social knowledge of the surroundings and transmit the data into a gateway, where the data is processed for the end-users [21,22,23,24,25,26]. The IoT technology ensures that the meaning significantly increases over the production activities within real-time monitoring, including its authority if connected by the wireless intelligent method instead of being generally identified as part of the wireless sensor networks (WSNs) [27].

In WSNs, information can be received by a single node, such as a humidity sensor node. Every sensor node inside the system can gather data about its surroundings to collect data [28]. The limited processing provides a capacity, so the nodes must play an economic determination role to obtain the confidence of its limitations from the overall. Hence, all sensor nodes within a network can possess an independent and sensible system, delivering resolutions via an onboard microcontroller [29]. LoRa signifies the entirety of alternate interaction modules that implement settings to machine-to-machine interfaces that are still external from the presence of networks such as 3G/4G [7,30]. A battery-based optimal communication environment has been similarly suggested as an optimal environment. The communication environment needs the least bat and most minor usage to convey a broadcast through the possibility of holding transmission-provided security [31,32,33,34,35].

More recent attention has focused on providing efficient LoRa communication system by deploying LoRa gateways to transfer the information to the remote devices. However, each sensor needs a single-hop LoRa gateway to ensure simultaneous communication because of the various obstacles between LoRa gateways and IoT sensors. A recent study developed wireless mesh networking for the continuous monitoring of IoT sensors, and their application was successful [4].

Most of the previous studies have focused on LoRa performance in scenarios such as single-hop, multi-hop, or hybrid combinations of wireless communication to establish a simultaneous and robust communication system for the devices.

While numerous research articles have been published in networking literature in recent years, very few researchers have addressed the issues of security, energy efficiency, and low-cost design and implementation of LoRa communication systems. For instance, studies [1,5,27] focused on cloud-based communications without elaborating on security and energy efficiency aspects.

The main objective of our study was to design and analyze a low-cost LoRa transceiver communication system that could effectively provide secure cloud-based services with good energy efficiency. We have conducted a thorough literature review from various credible outlets, including MDPI’s *sensors* and *electronics* journals, *Computer Communications* (Elsevier), *IEEE Access*, *IEEE Transactions on Instrumentations and Measurements*, and *IEEE Transactions on Vehicular Technology*. The summary of the related work on long-range transceivers is presented in Table 2. The main research contribution and year of publication are listed in Column 3 and 2, respectively. For each main contribution, we examined the aspects of security (low, moderate, and high), energy efficiency, cloud-based implementation, and system implementation cost. The security level, energy efficiency, cloud-based setup, and low-cost are listed in Column 4–7, respectively.

## 3. Methods: System Design and Analysis

**Figure 1:**
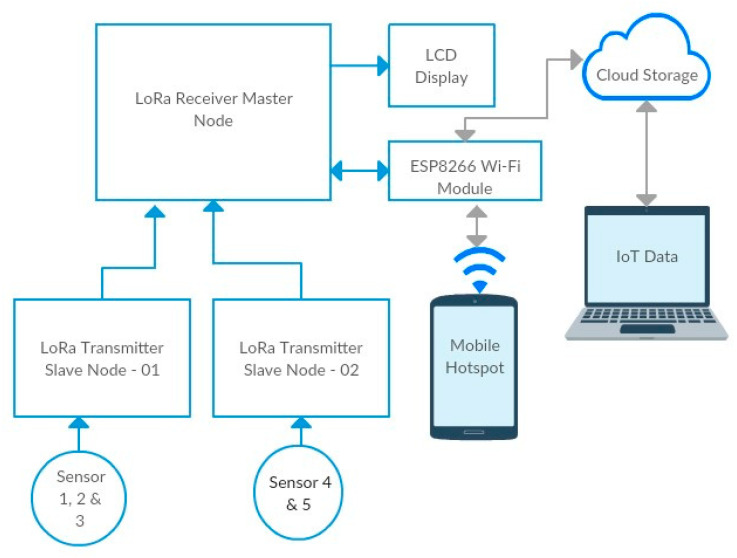
Block diagram of the proposed LoRa transceiver system.

The block diagram of the proposed LoRa transceiver system is shown in Figure 1. We designed and built (prototype) the interface unit, which includes the LoRa (Ra-02) communication module and a Wi-Fi Module. The hardware of the LoRa transceiver mainly consists of the ATMega328P Microcontroller, Nokia 5110 graphic LCD screen (Arduino Compatible), and Arduino Nano board. The sensors used in this study are the DS18B20 fire sensor, DHT11 temperature-humidity sensor, MQ-2 gas sensor, LM393 vibration sensor, and capacitive soil moisture sensor. The data from the IoT sensor nodes are transferred into the cloud using the Wi-Fi module in the transceiver system. The data are on an LCD display positioned beside the server and the gateway.

**Figure 2:**
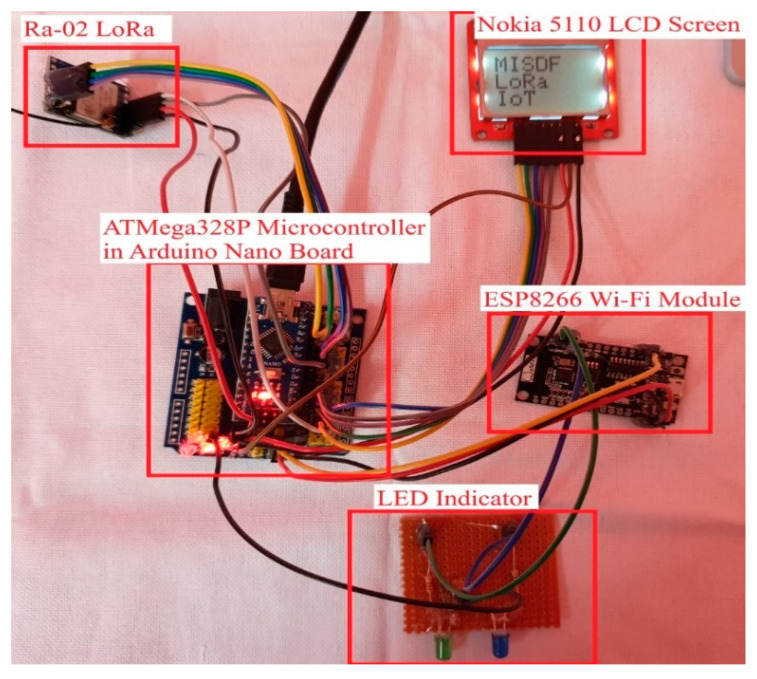
LoRa transceiver (prototype) with LCD screen and Wi-Fi module.

Figure 2 shows the LoRa transceiver unit, consisting of the master receiver node (Ra-02), an LCD display, and a Wi-Fi module. The Ra-02 LoRa is shown in the top left corner. The system was designed and built using two LoRa-enabled transmitter slave nodes and a master receiver node. The two transmitters (nodes) are connected to various IoT sensors that are suitable for indoor and outdoor conditions. The Ra-02 LoRa module is used for communications between transceiver nodes and the outside world. For instance, the Ra-02 receives IoT sensor data remotely. The data are pushed into the cloud using the Wi-Fi module. In addition, the receiver node is connected to an LCD screen for monitoring the IoT data.

The proposed system was built around a LoRa transceiver unit containing the ATMega328P Microcontroller, LCD screen, and an Arduino Nano board (Nokia, Japan). The sensors used in the device are the DS18B20 fire sensor, DHT11 temperature-humidity sensor, MQ-2 gas sensor, LM393 vibration sensor, and capacitive soil moisture sensor (Texas Instruments, USA). The data collected from the IoT sensor nodes are transferred into the cloud using a Wi-Fi module built on the system.

### 3.1. LoRa Parameters

The main LoRa parameters include the spreading factor, bandwidth, and code rate, which can be arranged by the data rate specifications, sensitivity, and communication range [23]. The LoRa signal strength ranges from −4 to 20 dBm, and the transmitted carrier frequency varies from 137 to 1020 MHz. In the proposed LoRa system, we use 433 MHz bands for transmissions. The LoRa manages the bandwidth of 125, 250, or 500 kHz. The high bandwidth allows for a higher data rate through digital signal processing. The high coding rate extends the presence in the air and increases the messages. The spreading factor (SF) allows several bits to be encoded at the respective symbol, ranging from 6 or 12. The increased SF raises the limit, increased SNR, and higher power consumption.

### 3.2. LoRa Master Receiver Node

We designed the master receiver node (Ra-02 LoRa) on a nano board controlled by the ATMega328P Microcontroller. An LCD screen is attached to the system for displaying the live sensor data. Moreover, the structure of the LoRa master receiver node is accomplished by including a Wi-Fi module to the board. This Wi-Fi module is a low-power unified Wi-Fi solution with a speed of up to 8 Mbps. It enables the receiver node to interface with the Internet and exchange the sensor data into the cloud.

The master receiver node is one of the main components of this LoRa transceiver system functioning as a beneficiary for receiving all sensor information from the transmitter nodes. The incoming sensor data are received in this master node with the assistance of the Ra-02 LoRa module. The Ra-02 module is an exceptionally viable device for receiving data from long distances without any Internet access. The data received in this node are managed by the microcontroller, and it displays the sensor data in the LCD screen appended to the board. The AVR architecture of the microcontroller has memory spaces, including data and program memory, to store and retrieve adequate information. The Wi-Fi module allows the master node to link to the outside world through the Internet; the Internet access assists the node in transferring the sensor data to cloud storage. The master node updates sensor data in the cloud storage within seconds by utilizing the accelerated Wi-Fi module.

The master receiver node and transmitter nodes are coded in the programming language C++. The coding methodology begins with the master receiver node. Two sets of instructions have been given to this node, one for the receiver and the other for the Wi-Fi module. After connecting the master node to the system, the board type, processor, and port must select the Arduino 1.8.9 IDE for transferring the code to board. The master node is coded as a receiver that collects the sensor information from all of the transmitter nodes. The Arduino software can be downloaded at https://www.arduino.cc/en/Main/Software (accessed on 30 Oct 2022). The software is installed in a workstation, and each node is connected to that system for uploading the instructions by means of a USB cable [29]. We verified the programming code before loading it to the master receiver node. After successfully programming the master node, only the power supply is required to run this node.

### 3.3. LoRa Transmitter Node 1

**Figure 3:**
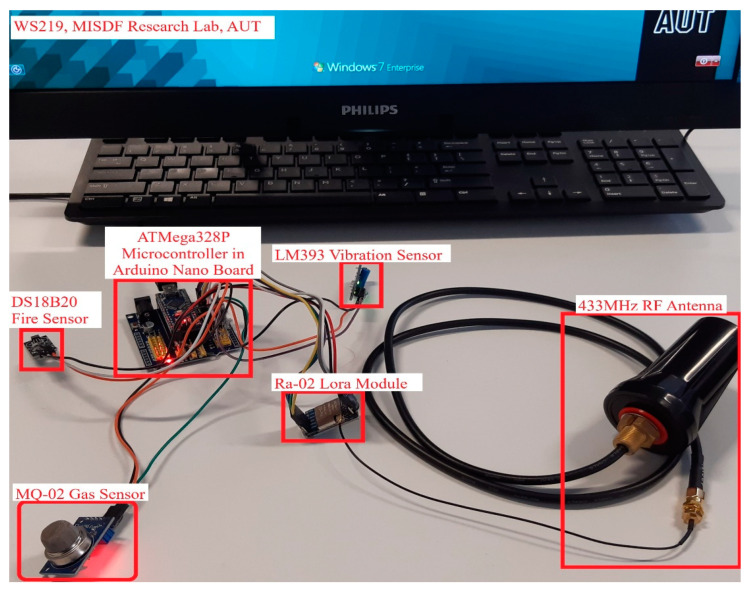
Illustrating the LoRa transmitter node–01 (prototype).

Figure 3 shows the LoRa transmitter slave node–01. The design and operation of the transmitter nodes of the LoRa Transceiver system are entirely different from the master receiver node. The primary function of the transmitter nodes is to collect the sensor data and transmit the information to the master node. The LoRa transmitter node–01 is structured by interfacing the hardware components of the microcontroller, Ra-02 LoRa Module, MQ-2 gas sensor, DS18B20 fire sensor, and LM393 vibration sensor on an Arduino Nano board. The association between the microcontroller and the Ra-02 Lora module is similar to the master node, yet the working of the device is distinctive. In this node, the Ra-02 LoRa module acts as a transmitter that only sends the sensor information to the master node.

There are three sensors linked with this node, and each sensor performs its own operation. The performance of the LoRa transceiver system must be analyzed both indoor and outdoor conditions; therefore, three sensors have been deployed for internal monitoring. This node is intended for detecting fire, gas, and vibrations inside the building. The fire sensor utilized for this node is the DS18B20 digital thermometer, which gives 9-bit–12-bit Celsius temperature estimations and performs well with any sort of microcontroller. It can withstand a temperature range between −55 and +125 °C and also has a user-programmable alarm system. The MQ-2 gas sensor is the most competent sensor for distinguishing any sort of gas spillage. The quick response time and high sensitivity of this equipment is appropriate for detecting alcohol, smoke, and LPG. An onboard potentiometer in the LM393 vibration sensor can be adjustable by the user-defined threshold level of the device to identify the vibrations. These extraordinarily intelligent sensors attached to the transmitter node–01 provide accurate sensor data, and the information will be rapidly processed and instantly transmitted to the master node with the backing of Ra-02 LoRa module.

### 3.4. LoRa Transmitter Node 2

**Figure 4:**
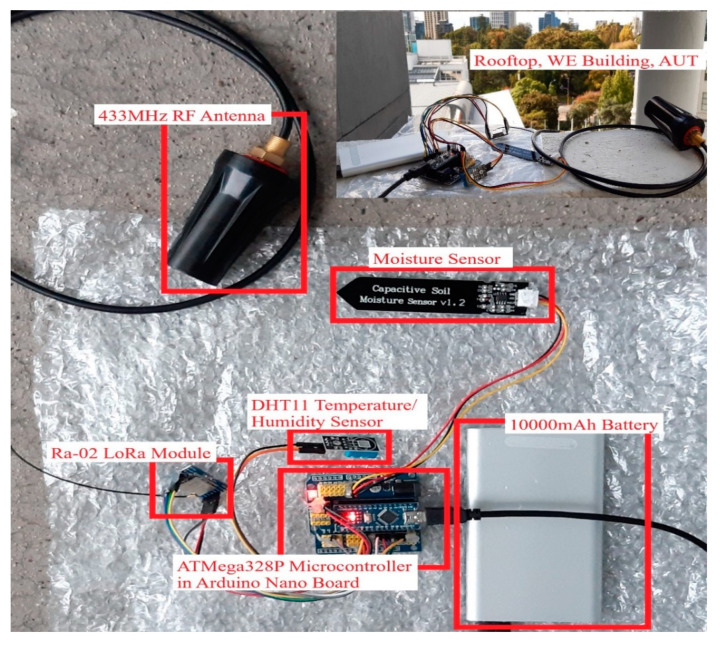
Deployment of LoRa transmitter node–02 at the rooftop of the AUT building.

Figure 4 shows the deployment of the LoRa transmitter node–02 at the rooftop of the University building. An additional transmitter node–02 was designed to assess the productivity of the transceiver system in open-air conditions. The structure of the transmitter node–02 is practically the same as transmitter node–01, and the progressions made for this node is the utilization of different sensors. In transmitter node–02, the sensors that are associated with the microcontroller are the DHT11 temperature-humidity sensor and capacitive soil moisture sensor. The considerable achievement of the temperature-humidity sensor is that it can refresh the reading every 2 s. This low-cost, efficient sensor conveys a digital signal on the data pin. The capacitive soil moisture sensor provides information on the volumetric water content in the soil and is compatible with any type of microcontroller.

The sensors fixed in this node are reasonable for investigating the performance of the LoRa transceiver system in outdoor conditions. The functioning of this node is similar to that of the transmitter node–01. The sensors affixed to this node collect the digital data of the outdoor conditions such as moisture, temperature, and humidity. At that point, it exchanges the information to the master receiver node at a long distance by means of the Ra-02 LoRa module.

The performance of the LoRa transmitter node–02 has been tested both in the lab and in outdoor environments. The maximum range accomplished by this node was 10 km, and the sensor data was updated within 1 s. The DHT11 temperature-humidity sensor and capacitive soil moisture sensor are fixed in this node to evaluate the performance of the LoRa transceiver system. The 433 MHz water-resistant RF antenna relates to this node to transmit signals in rainy conditions. We tested the performance of this node using sensor data collected.

### 3.5. Programming the Transmitter Node

The transmitter node–01 is programmed to act as the transmitter that transmits the sensor data to the master node. The significant sections and functions that are utilized in the transmitter node–01 coding include all the required libraries at first. At that point, the sensors connected in the node are characterized by a pinout setup, and the frequency of the LoRa transceiver system of 433 MHz is also defined in the code. The transmitter node–02 is programmed similar to that of node-01. The focus is to characterize the sensors (e.g., Temperature, Humidity, Moisture) in the code.

### 3.6. Wi-Fi Module Configuration

The ESP8266-based Wi-Fi module facilitates an Internet connection to the master receiver node for transmitting the sensor data into the cloud for storage. We develop and configure a low-cost Wi-Fi module to be used with a microcontroller with an integrated TCP/IP protocol stack. The multifunctional Wi-Fi module can act as an access point (AP) to form a Wi-Fi hotspot. The system is programmed using C++ and configured for optimum performance.

## 4. Methodology

The master receiver node is one of the main components of the proposed LoRa transceiver system. It operates on a frequency of 433 MHz. The input power supply to this node is 7–12 V, and the power consumption is 9 mA. In the experiments, two transmitter nodes were used to evaluate the performance of the LoRa transceiver at various locations. We first evaluated the performance of LoRa transmitter slave node–01 in the laboratory at Auckland University of Technology (AUT). We also evaluated the performance of the LoRa master receiver node in the laboratory. Next, we then tested the performance of LoRa transmitter node–02 in the outdoor environment to distinguish temperature, humidity, and moisture.

Finally, the LoRa transceiver system was tested for distance coverage by placing the master node on the rooftop of AUT’s Tower building. This site was selected as a primary location suitable for testing the node against wind, rain, temperature, and moisture. The secondary location was Liston Park, which is about 10 km away from the primary location (AUT). A power bank (10,000 mAh) was used to operate the LoRa system during the field experiments.

## 5. Results and Discussion

The results from the field trials show that the proposed LoRa system can be used for a network coverage of about 10km. The spreading factor holds some notable influence on the network coverage and data transmission rate. The system serves the purpose of achieving low-power consumption over long-distance coverage. The system also displays accuracy through LCD screen alterations from specific demands that can be tracked in the future. It is an IoT and LoRa wireless module that promotes consecutive secure monitoring applications. We verified and analyzed the output data collected from the various sensors using an LCD monitor. The data accessibility from the cloud and data synchronization at regular intervals were also verified.

### 5.1. Real-Time IoT Data Monitoring and Storage

The results from the field trial measurements were obtained in two ways. First, we monitored real-time IoT sensor data by connecting the LoRa master receiver node to an LCD screen. Second, we accessed the IoT sensor data in the cloud. For system performance testing, a database was created in the cloud, and IoT sensor data were transferred into the database using the ESP8266 Wi-Fi module. The verification process involved storing IoT sensor data (received from the transmitter nodes) in the cloud database. This sensor data was stored in the appropriate tables and columns for each sensor node, ensuring that the cloud storage was functioning. We also tested the system performance in various climatic and distance conditions. The IoT sensor data was monitored by an LCD unit at the receiver node. Finally, we successfully uploaded IoT sensor data into the cloud.

### 5.2. Data Access in the Cloud

The IoT sensor data is automatically updated in the cloud that verifies the storage. The test results proved that the sensor data can easily be accessible from cloud storage using an Internet-enabled device such as a computer, laptop, or smartphone. The LoRa node1 displays the information from the transmitter node–01 that consists of fire, vibration, and gas sensor data. Another link for LoRa node2 shows the information of the transmitter node–02, which contains the sensor information of temperature, humidity, and moisture. An Internet connection is required to access the sensor data of both nodes from anywhere in the world. The monitoring and controlling of the LoRa-enabled sensors from any remote location is a dynamic achievement of this experimental work.

### 5.3. Efficiency and Data Accuracy

**Figure 5:**
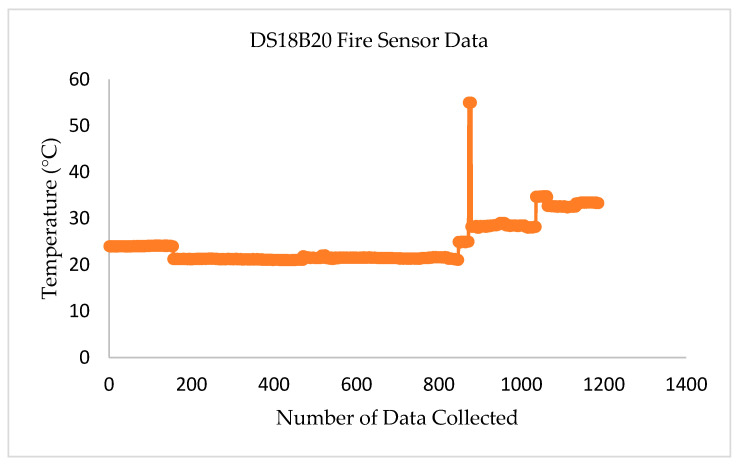
Test results for DS18B20 fire sensor.

Figure 5 shows the laboratory test results for the DS18B20 Fire Sensor. More than 1000 data sets were collected from every sensor node to evaluate the time efficiency and data accuracy of the transceiver system. The fire sensor in transmitter node–01 delivers the temperature (measured in Celsius). If the temperature inside the building abnormally rises, a warning sound is activated in the sensor to notify the incident. The collected data indicate that this sensor provides the ordinary room temperature of 20 to 25 °C. For testing the accuracy and response of the sensor, a manual flame was placed near the sensor, and the temperature level was recorded at about 55 °C.

**Figure 6:**
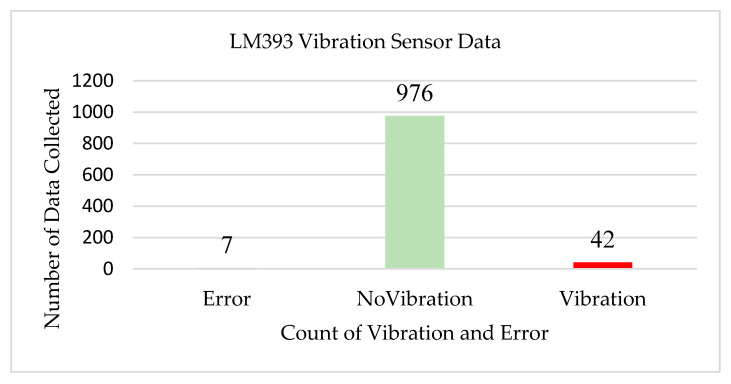
Test results for LM393 vibration sensor.

Figure 6 shows the data accumulated from the vibration sensor (LM393). The LM393 vibration sensor in transmitter node1 is utilized to recognize the vibrations over a threshold point. For evaluating the performance and accuracy of the vibration sensor, 1025 stored sensor data sets were extracted from the cloud. The sensor counted 976 times as ‘No Vibration’, and the vibrations happened 42 times at a specific time. An error reading was also recorded seven times. Hence, the accuracy rate of this sensor in transmitter node1 was determined as 99.31%, and the error rate was 0.68%.

**Figure 7:**
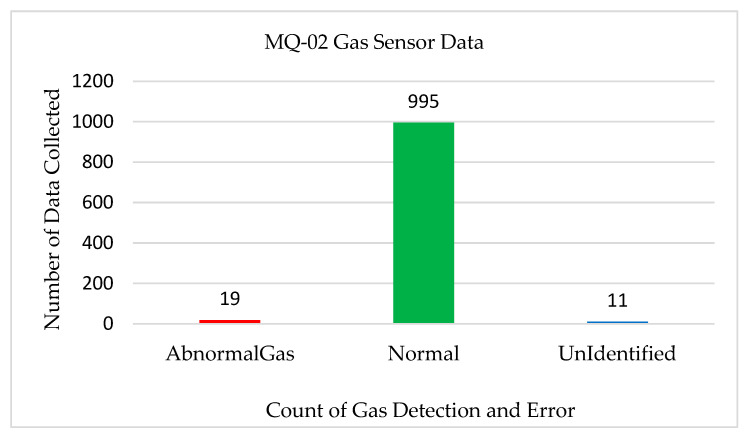
Test results for the MQ-2 gas sensor.

Figure 7 shows the test results for the gas sensor. To analyze the system efficiency, we recorded 1025 sensor data sets using the MQ-2 gas sensor. We manually inserted smoke and gases into the system and performed various tests for system accuracy. We observed that for 1025 sensor data sets, the system identified 19 instances of abnormal gases and 11 cases of no gases. The system accuracy was found to be 99% with an error rate of 1%.

**Figure 8:**
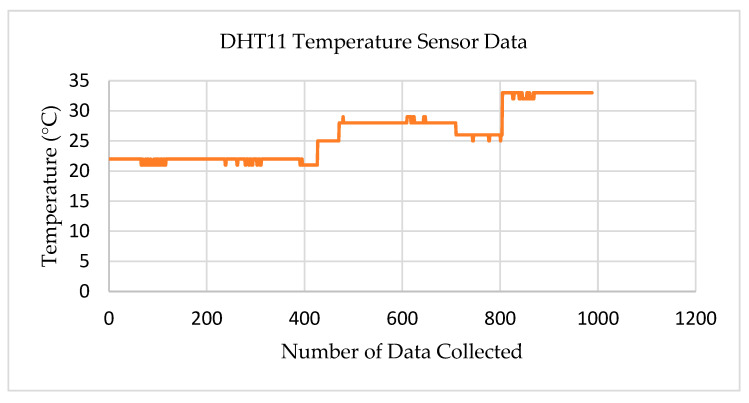
Test results for the DHT11 temperature sensor.

Figure 8 shows the test results for the DHT11 temperature sensor. We deployed temperature sensors in Liston Park (Auckland), which is about 10 km away from Auckland City. The transmitter node was installed at the rooftop of a seven-story building. The first 500 data points distinguished the temperature range from 20 to 25 °C at the Liston Park, and the remaining data at a temperature ranging from 25 to 33 °C at the rooftop of the AUT building. Hence, the temperature sensor in the LoRa transceiver system could successfully transmit IoT sensor data accurately up to a distance of 10 km.

**Figure 9:**
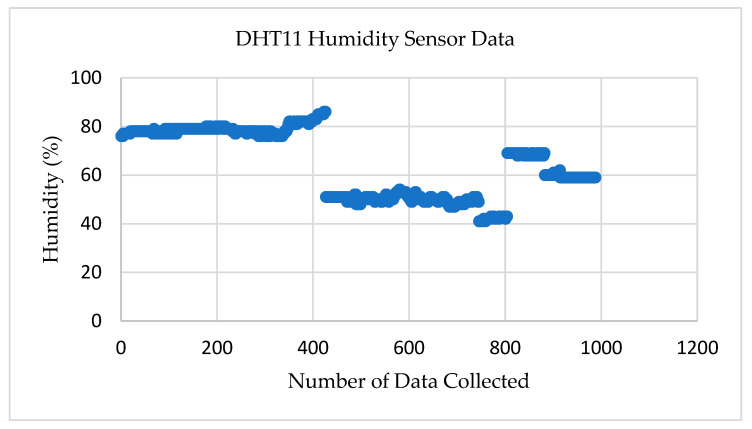
Test results for the DHT11 humidity Sensor.

Figure 9 shows the test results for the DHT11 humidity sensor. The humidity sensor is also part of the temperature sensor in transmitter node–02, but this sensor delivers the atmospheric humidity level. The sensor measures the relative humidity (RH) in percentage by calculating the amount of water content present in the atmosphere. The data were collected by placing the transmitter node–02 in various locations. The sensor reading showed that the dissimilarity in the values according to the location change.

At first, the data were gathered from the sensor by locating the node in Liston Park, Ellerslie, Auckland. The humidity level was recorded as 70–80% because of the environmental condition in the park. Later, the node was placed at the rooftop of the AUT building, where the humidity level was 40–60%. The sensor accurately transferred sensor data from both locations.

**Figure 10:**
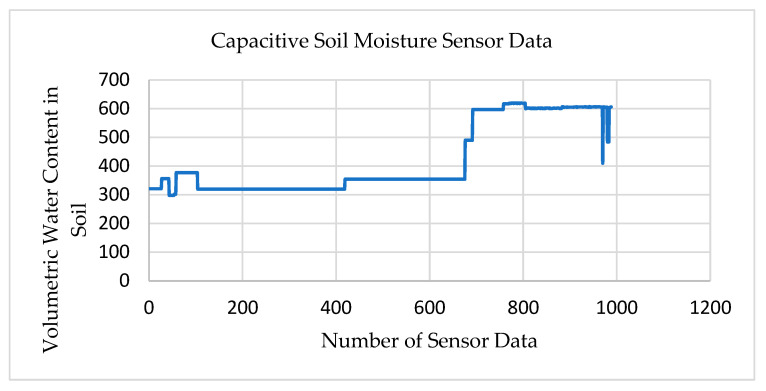
Test results for the soil moisture sensor.

Figure 10 shows the data collected from the soil moisture sensor. The capacitive soil moisture sensor is another sensor included in the transmitter node–02. This sensor measures the volumetric water content in the soil.

The moisture level in the soil is categorized into three levels to quickly distinguish the condition of the soil. A moisture level of 260–350 indicates high water content in the soil, and 350–430 indicates the normal wet condition. However, moisture level 430–520 shows that the soil condition is dry. The initial 600 sensor data was accumulated from the Liston Park, Auckland, and the moisture level in the soil varied from 300 to 400. Then, the transmitter node–02 was placed in the rooftop of the AUT building and obtained a moisture level of 500, indicating no moisture at all. We observed a variation in the results, which indicated that the transmitter nodes were accurately functioning at a distance of up to 10 km in diverse meteorological conditions. Therefore, the sensors attached in transmitter node–02 can be used to effectively transmit data to the master node over 10 km.

### 5.4. Transmitter Efficiency Test Results

The time efficiency of the transmitter node was scrutinized by giving manual inputs to the sensors. The response time of the sensor was observed to determine the efficiency of the transmitter node. Table 3 shows the time efficiency of the accumulated sensor data variations with date and time. This field experiment was conducted in the laboratory at AUT.

We observed the efficiency of the vibration sensor at recognizing the vibrations and idle state of the sensor and transmitting the data to the master node within seconds. The results indicated a variation in the sensor data being updated to the master node in the range of 1–20 s.

Table 4 shows the time efficiency of the node to report variations in the gas sensor. The MQ-2 gas sensor data were accumulated over several days at various locations to determine the efficiency of the node. The sensor data accurately updated in the cloud within 10–20 s. Both transmitter nodes were examined in dissimilar conditions for evaluating the efficiency, accuracy, and speed of the data transfer. The results show that the IoT sensor data were successfully transferred from the transmitter to the receiver (10 km) within a few seconds. In summary, our proposed LoRa transreciver system provides accurate sensor data in linking city and suburban communities. The cloud deployment in the transceiver system allows us to access the sensor data anytime and anywhere.

### 5.5. Practical Implications

The results presented in Section 5 provide some insights into the practical implementation aspect of the LoRa transceiver System. This research provides a solution to the problems that smart homes and cities have in connecting to 250 sensor nodes through a single gateway. This research also provides a clear perception of designing a secure low-cost LoRa transceiver system. The field experiments prove that this system can be used to link city and suburban communities covering about 10 km at no cost (no need to go through service providers). The spreading factor holds some notable influence on the network coverage and data transmission rate. The system serves the purpose of achieving a low-power consumption over a long-distance coverage. The system also displays real-time IoT data through an LCD screen. In this research, we developed, configured, and tested the LoRa transceiver, Wi-Fi module, and LCD display unit in the laboratory, and found the setup to be robust. This research can be taken into the next step for commercialization as well as production.

## 6. Concluding Remarks

A LoRa transceiver system with two transmitter nodes has been designed and built in the laboratory at a cost of about USD 150, which can be used for the monitoring and storing of IoT sensor data in the cloud. We also provided a solution for sending and retrieving IoT data to and from the cloud by designing and configuring a Wi-Fi module. The system performance was tested (both indoor and outdoor conditions) using field data and was found to be robust. The results obtained have shown that the transmitter nodes perform well for up to 10 km, and the receiving data accuracy is found to be 99%. The reaction time of the transmitter nodes is determined by the sensor data accumulation as within 2–20 s. The system is noticeably time-efficient and provides accurate sensor data effectively.

The future expansion of our LoRa transceiver system is also possible through the implementation of an image sensor in the transmitter node to transmit photographs of the incident or even provide a livestream of the climate conditions. Designing a robust software module to facilitate the detection and retransmission of dropped packets is also suggested as a future work.

## Figures and Tables

**Table 1:** List of abbreviations used in this paper.

Abbreviation	Definition	Abbreviation	Definition
IoT	Internet of Things	3G	Third Generation Cellular network
IP	Internet Protocol	GPS	Global Positioning System
LCD	Liquid Crystal Display	WSN	Wireless Sensor Network
LED	Light-Emitting Diode	PV	Photovoltaic
LoRa	Long-Range	DHT	Digital Temperature Humidity
LPWAN	Low-Power WAN	PLC	Power Line Communication
LoRaWAN	Long-Range WAN	IDE	Integrated Development Environment
LTE	Long-Term Evolution	GUI	Graphical User Interface
M2M	Machine-to-Machine	AQI	Air Quality Index
MAC	Media Access Control	IAQ	Indoor Air Quality
MIC	Message Integrity Code	LPG	Liquefied Petroleum Gas
NBIoT	Narrow-Band IoT	EnMoS	Environmental Monitoring System

**Table 2:** Summary of the related work on long-range transceivers.

Reference	Year	Main Contribution	Security Level	Energy Efficient?	Cloud-Based?	Low-Cost?
[4]	2018	Developed a mesh network framework for monitoring IoT applications.	Moderate	Yes	Yes	No
[28]	2018	Designed a LoRa transceiver system with improved characteristics.	Low	Yes	No	No
[36]	2018	Focused on an automatic key generation for long-range wide area communication.	High	Yes	No	No
[31]	2018	Validated LoRa experimentation using commercial devices and software-defined radios.	Low	No	No	No
[37]	2018	Developed a narrowband interference suppression technique to improve BER.	Moderate	Yes	No	No
[22]	2018	Developed an energy-efficient network architecture for IoT.	Low	No	Yes	Yes
[38]	2018	Developed a scheme for overcoming the bandwidth limitation of LoRa.	High	Yes	No	No
[39]	2017	Developed a multi-hop network based on low-power wide area technology.	Low	No	No	No
[12]	2017	Proposed an architecture of IoT LoRa wireless radio-based information display system.	Moderate	Yes	No	Yes
[1]	2022	LoRa communication system for the development and implementation of a smart multi-sensor system for monitoring air quality remotely.	Low	Yes	Yes	No
[5]	2022	LoRaWAN technique to provide a low-power livestock localization and monitoring system	Moderate	No	Yes	Yes
Our work	A low-cost secure system for monitoring and storing of IoT data in the cloud that can be used to link city and suburban park communities effectively.		High	Yes	Yes	Yes

**Table 3:** Time efficiency of the vibration sensor in transmitter node–01.

Fire	Vibration	Gas	Date	Time
28.44	No Vibration	Normal	11 April 2019	1:08:59 a.m.
28.37	Vibration	Normal	11 April 2019	1:08:58 a.m.
28.37	Vibration	Normal	11 April 2019	1:08:50 a.m.
28.37	Vibration	Normal	11 April 2019	1:08:49 a.m.
28.37	Vibration	Normal	11 April 2019	1:08:48 a.m.
28.37	Vibration	Normal	11 April 2019	1:08:43 a.m.
28.37	No Vibration	Normal	11 April 2019	1:08:17 a.m.
28.44	No Vibration	Normal	11 April 2019	1:06:27 a.m.
28.5	Vibration	Normal	11 April 2019	1:06:15 a.m.
28.5	Vibration	Normal	11 April 2019	1:06:14 a.m.
28.5	Vibration	Normal	11 April 2019	1:06:13 a.m.
28.5	Vibration	Normal	11 April 2019	1:06:11 a.m.
28.5	Vibration	Normal	11 April 2019	1:06:10 a.m.

**Table 4:** Time efficiency of the gas sensor in transmitter node-01.

Fire	Vibration	Gas	Date	Time
21.12	NoVibration	Normal	27 April 2019	11:24:14 a.m.
21.06	NoVibration	AbnormalGas	27 April 2019	11:23:58 a.m.
21.06	NoVibration	AbnormalGas	27 April 2019	11:23:33 a.m.
21.06	NoVibration	AbnormalGas	27 April 2019	11:23:22 a.m.
24.94	NoVibration	Normal	23 April 2019	4:00:53 a.m.
28.37	NoVibration	Normal	11 April 2019	12:56:42 p.m.
28.37	NoVibration	AbnormalGas	11 April 2019	12:56:32 p.m.
28.31	NoVibration	Normal	11 April 2019	12:56:17 p.m.

## Data Availability

Not applicable.
